# Cyclosporin A: A Repurposable Drug in the Treatment of COVID-19?

**DOI:** 10.3389/fmed.2021.663708

**Published:** 2021-09-06

**Authors:** Christian A. Devaux, Cléa Melenotte, Marie-Dominique Piercecchi-Marti, Clémence Delteil, Didier Raoult

**Affiliations:** ^1^Aix-Marseille Univ, IRD, APHM, MEPHI, IHU-Méditerranée Infection, Marseille, France; ^2^CNRS, Marseille, France; ^3^Department of Legal Medicine, Hôpital de la Timone, Marseille University Hospital Center, Marseille, France; ^4^Aix Marseille Univ, CNRS, EFS, ADES, Marseille, France

**Keywords:** SARS-CoV-2, COVID-19, cyclosporin A, cyclophilin, angiotensin converting enzyme-2

## Abstract

Coronavirus disease 2019 (COVID-19) is now at the forefront of major health challenge faced globally, creating an urgent need for safe and efficient therapeutic strategies. Given the high attrition rates, high costs, and quite slow development of drug discovery, repurposing of known FDA-approved molecules is increasingly becoming an attractive issue in order to quickly find molecules capable of preventing and/or curing COVID-19 patients. Cyclosporin A (CsA), a common anti-rejection drug widely used in transplantation, has recently been shown to exhibit substantial anti-SARS-CoV-2 antiviral activity and anti-COVID-19 effect. Here, we review the molecular mechanisms of action of CsA in order to highlight why this molecule seems to be an interesting candidate for the therapeutic management of COVID-19 patients. We conclude that CsA could have at least three major targets in COVID-19 patients: (i) an anti-inflammatory effect reducing the production of proinflammatory cytokines, (ii) an antiviral effect preventing the formation of the viral RNA synthesis complex, and (iii) an effect on tissue damage and thrombosis by acting against the deleterious action of angiotensin II. Several preliminary CsA clinical trials performed on COVID-19 patients report lower incidence of death and suggest that this strategy should be investigated further in order to assess in which context the benefit/risk ratio of repurposing CsA as first-line therapy in COVID-19 is the most favorable.

## Introduction

The first outbreak of coronavirus disease 2019 (COVID-19) was reported by China at the end of 2019 (1–3). Evidence was rapidly reported that patients were infected by a novel betacoronavirus lineage 2b/sarbecovirus tentatively named 2019 novel coronavirus (2019-nCoV) before being known as severe acute respiratory syndrome coronavirus 2 (SARS-CoV-2) with respect to its phylogenetic relationship (80% nucleotide identity) with the SARS-CoV (4). To date, it is the seventh characterized coronavirus described as capable of causing a respiratory infection in human. From the start of 2020, COVID-19 has become a global pandemic and has been declared a global health emergency by the World Health Organization (WHO). In 1 year, more than 75 million people were infected worldwide and this virus has caused more than 1.6 million deaths (https://coronavirus.jhu.edu/map.html, December 18, 2020). Depending on the health status, age, and comorbidities (hypertension, coronary heart diseases, cerebrovascular diseases, diabetes, chronic kidney diseases) of the infected individuals, SARS-CoV-2 may either be asymptomatic, give a picture of influenza infection, or induce severe forms of COVID-19 with acute respiratory distress syndrome and multiple organ failure syndrome which can lead to death in about 2.27% of infected individuals (2, 5, 6).

The SARS-CoV-2 is an enveloped RNA^+^ virus surrounded by spike (S) glycoproteins. The genomic length of SARS-CoV-2 is about 30 kb and encodes as many as 14 open reading frames (ORFs) leading to the synthesis of 29 proteins (7, 8). Coronaviruses have the largest viral RNA genomes known to date (e.g., human immunodeficiency virus genome is only 10 kb), and it was hypothesized that their expansion and selection was likely enabled by acquiring enzyme functions that counter the high error frequency of RNA polymerases (9). During the early infection process, the trimeric SARS-CoV-2 S1 spike first binds to the N-terminal portion of the angiotensin I-converting enzyme 2 (ACE2) which acts as viral receptor at the surface of susceptible cells (10). In addition to ACE2, molecules such as neuropilin-1 (11), chaperone GRP78 (12), and CD209/DC-SIGN (13) can act as SARS-CoV-2 receptors or co-receptors. Furthermore, the cellular transmembrane protease serine 2 (TMPRSS2) contributes to enhance the S-protein-driven viral entry (14). After cleavage at the S1/S2 junction, the S2 takes the conformation required for insertion of the fusion peptide into the cellular lipid bilayers. The viral nucleocapsid is thus delivered into the cytoplasm through the endocytic vesicle. After acidification of the late endosome, the action of cathepsin enables the uncoating of the genomic RNA. SARS-CoV-2 like other pathogenic CoVs possesses a linear plus-sense strand RNA genome (gRNA) that has a 5′ methylated cap and 3′ poly-A tail, allowing its anchorage to ribosomes for the synthesis of polyprotein precursor. The two-thirds of this gRNA (about 20 Kb) is occupied by the ORF1a (expressed by genome translation) and ORF1ab (expressed by genome translation and ribosomal frameshift) and encodes the polyproteins precursors pp1a and pp1ab, respectively, giving rise to the production of 16 non-structural proteins (Nsps) by auto-proteolytic processing (15–17). The 3′-proximal third sequence of the gRNA serves as template for several subgenomic mRNAs having common 3′ UTRs (18) that encode the viral structural (the spike/S, the envelope/E, the membrane/M, and the nucleocapsid/N) and accessory proteins. The S, E, and M proteins are synthesized and anchored on the endoplasmic reticulum (ER) with the N protein translated in the cytosol. Post-translational modifications of viral proteins occur within the endoplasmic reticulum and trans-Golgi network vesicles. After assembly in the ER–Golgi intermediate compartment (ERGIC), the E protein plays an essential role in virus assembly and the mature M protein shapes the virus. Mature virions are released from smooth-walled vesicles by exocytosis. The accumulation of knowledge relating to the intracellular cycle of replication of the virus as well as the nature of the interactions between the viral and cellular proteins is essential to choose in the large panel of FDA-approved therapeutic compound the molecules capable of blocking the deleterious effects of this virus in infected individuals or to design new antiviral drugs.

Because of the urgent need for safe and efficient therapeutic drugs able to lower morbidity and mortality of COVID-19, multiple clinical trials have been conducted including repurposing of antiviral drugs, anti-inflammatory molecules, and also all kinds of low-cost old drugs known for their *in vitro* antiviral properties. Several independent studies reported in the literature had revealed the *in vitro* antiviral properties of cyclosporin A (CsA), a well-characterized immunosuppressant largely used in the prevention of graft rejection. *In vitro*, this drug was shown to be active against different viruses and to inhibit the replication of coronaviruses, including that of HCoV-229E and SARS-CoV-1 (19, 20). Unsurprisingly, when tested *in vitro* on SARS-CoV-2, CsA was also found to inhibit the replication of this new virus (21). Moreover, the CsA analog alisporivir (called Debio-025) was also shown to block SARS-CoV-2 replication *in vitro* (22, 23). The question of CsA or CsA analog use in the treatment of COVID-19 is now more pressing (Table 1).

**Table 1:** *In vitro* activity of cyclosporine A against viruses.

**Virus**	**Cyclophilin inhibitor**	**Read out**	**Dose of action**	**Effect**	**References**
SARS-CoV-2	Cysclosporin A	Vero E6 cells model of SARS-CoV-2 infection	IC_50_: 3 μM	Reduce viral production	(21)
SARS-CoV-2	Debio-025	Vero E6 cells	0.46 ± 0.04 μM	Reduced SARS-CoV-2 RNA production in a dose-dependent manner	(22)
SARS-CoV-2	Debio-025	Vero E6 cells	4.3 μM	Reduced SARS-CoV-2 progeny virions production	(23)
SARS-CoV-1	Cysclosporin A	Vero E6 cells and 293/ACE2 cells.	16 μM	Reduced viral replication and reporter gene expression of SARS-CoV–GFP; inhibition of SARS-CoV RNA synthesis; the protein synthesis was almost undetectable	(19)
SARS-CoV-1	Debio-025	Vero E6 cells	4.3 μM	Reduced SARS-CoV progeny virions production	(23)
SARS-CoV-1	FK506	VeroFM cells	EC_50_: 6.9 μM	Decreased viral infection and inhibition of SARS-CoV-1 replication	(24)
HCoV- 229	Cysclosporin A	Huh7 cells	32 μ	Reduced reporter gene expression and the production of infectious progeny were also significantly decreased	(19)
HCoV-229E	FK506	HuH7 cells	EC_50_: 5.4 μM	Decreased viral infection and inhibition of HCoV-229E replication	(24)
HCoV-NL63	FK506	CaCo2 cells	EC_50_ of about 13.4 M	Decrease viral infection and inhibition of HcoV-NL63replication	(24)
Human immunodeficiency virus type 1 (HIV-1)	Cysclosporin A	Human CD4^+^ T cells Jurkat target cells	2.5 μM 2.5 μM	Reduced viral infectivity	(25)
HIV-1	Cysclosporin A	Jurkat T cells	10 μM	Decreases gp120^env^ and gp41^env^ incorporation into HIV-1 virions and impaired fusion of these virions with susceptible target cells	(26)
HIV-1 (HIV-1 _NL4−3_)	Cysclosporin A	HIV Rev-dependent indicator cell line and Peripheral blood mononuclear cells (PBMCs)	All dosage s from 100 to 600 nM	Inhibits HIV-1 replication (including subtherapeutic concentrations)	(27)
HIV-1	SDZ NIM 811	MT4 cell line (human T-cell leukemia virus-transformed T4 cell line)	IC_50_: 0.084 g/ml	Inhibits HIV-1 replication	(28)
HIV-1	STG-175	Peripheral blood mononuclear cells (PBMCs)	0.5 and 5 μM	Inhibits HIV-1 replication	(29)
HIV-1 (HIV-1_LAI_)	FK506-modified HIV-protease inhibitor	T cells	IC_50_ of 4.2 nM	The FK506-modified HIV-protease inhibitor retains anti-HIV-1 protease Activity *in vitro* and is partitioned into the cellular component of whole blood via binding to FKBP	(30)
HIV-1	Cyclophilin Inhibitor CPI-431-32	Blood-derived CD4^+^ T-lymphocytes	2 μM	Inhibits HIV-1 replication	(31)
Hepatitis B virus (HBV)	Cysclosporin A	HepaRG; HepAD38; primary human hepatocytes primary human hepato-cytes	4 μM	Inhibits HBV entry into cultured hepatocytes decreased HBs and HBe secreted from the infected cells in a dose-dependent manner decreased HBs and HBe secreted from the infected cells in a dose-dependent manner CsA decreased HBs and HBe secreted from the infected cells in a dose-dependent manner (Inhibits the transporter activity of sodium taurocholate cotransporting polypeptide, NTCP)	(32)
HBV	STG-175	Human hepatoma Huh7.5.1 cells	0.5 and 5 μM	Decreased HBV replication	(29)
Hepatitis C virus (HCV)	Cysclosporin A	Huh 5-2 cells	EC_50_: 2.8 ± 0.4 μg/mL	Inhibition of HCV subgenomic replicons	(33)
HCV	Debio-025 in combination with other antiviral drugs	Hepatoma cells	0.1 or 0.5 μM	Antiviral activity in short-term antiviral assays	(23)
HCV	NIM811	Huh7 cells	1–3 μg/ml	Reduction of HCV RNA levels	(34)
HCV	NIM811	Huh 21-5 cells	IC_50_: 0.66 μM	Reduction of HCV RNA levels	(35)
HCV	SCY-635	MDCKII-hMDR1 cells	IC 50: 0.20 μM	Inhibition of HCV replication	(36)
HCV	STG-175	Human hepatoma Huh7.5.1 cells	0.5 and 5 μM	HCV cell clearance	(29)
HCV	Cyclophilin inhibitor CPI-431-32	Human hepatoma Huh7.5.1 cells	2 nM	Inhibition of HCV replication	(31)
Mouse hepatitis virus (MHV)–GFP	Cysclosporin A	17CL1 cells	16 μM	Reduction of reporter gene expression and progeny virions	(19)
Vesicular stomatitis virus (VSV)	Cysclosporin A	BHK cells	25 mM	Inhibition of VSV-NJ infectivity	(37)
Flaviviruses (including West Nile virus, dengue virus, yellow fever virus)	Cysclosporin A	Huh-7.5 cells	8-20 μM	Reduced viral RNA synthesis and flavivirus production	(38)

## Discovery of Cyclosporin A, a Cyclophilin Inhibitor, and FK506, an FKBP Inhibitor

The cyclosporin story started in the 1969–1970 at the Sandoz Laboratories in Basel (Switzerland). The 11-amino-acid lipophilic cyclic peptide cyclosporin (CsA, also known as ciclosporin) of 1.2 kDa molecular weight, produced from the fungus *Tolypocladium inflatum* and other microorganisms such as *Fusarium solani, Neocosmospora varinfecta*, and *Aspergillus terreus* (39), was found to exhibit immunosuppressive properties offering new hope to transplant surgeons to avoid transplant rejection of the patients. The CsA cyclic peptide is insoluble in water and soluble in ethanol or in olive or sesame oil at 60°C and next can be kept in a solution at room temperature. The olive oil-soluble form of the peptide supplemented with 12.5% ethanol was the first form of manufactured CsA for oral administration, which must be dispersed in juice or milk for ingestion (40). CsA was introduced in clinical practice in 1978 (41). The bioavailability of the original corn oil-based preparation of cylosporine (Sandimmune^®^, Novartis Pharma, Basel, Switzerland) largely varied in cyclosporine blood levels among patients leading to the development of microemulsion formulation (Neoral^®^, Novartis Pharma) (42, 43). Usually, a dose of 20 mg CsA/kg daily is recommended after solid organ transplant with progressive decrease every week down to 5 mg/kg daily, while a dose of 1 mg/kg daily is recommended after hematopoietic stem cell transplantation (44). Upon administration, CsA is absorbed at the intestinal level by the epithelial cells and the efficiency of this process is influenced by different factors such as dietary composition or bile flow. In the plasma, CsA is found bound to lipoproteins and spreads in the extravascular space (45). CsA is metabolized by liver cells through the P450 3A4 (CYP3A4) leading to the generation of a number of metabolites (46). After a single dose of CsA, there is a peak of drug blood concentrations (Cmax) during the first 2 h followed by elimination (C0), and the drug bioavailability should be carefully monitored in clinical settings using the Cmax and a measure of drug concentration every 2 h (C0, C2, C4, C6, C8) to determine when an additional dose should be administered (47).

The mechanism of action of CsA was elucidated in 1984 with isolation from thymocytes of cyclophilin (CyP), an 18-kDa highly basic charged cytosolic protein that binds CsA with high affinity (48). Next, a structurally different immunosuppressant, a macrolide named FK506 isolated from *Streptomyces tsukubaensis*, emerged and was found to interfere with T-cell activation through a similar mode of action than CsA leading to suppression of mixed lymphocyte reaction (MLR), interleukin (IL)-2 and IL-2 receptor, IL-3, and γ-interferon (49). Like CsA, FK506 binds to a member of peptidylproline cis-trans isomerase (PPIase) family, but instead of binding cyclophilin (also called rotamase), it binds the FK506-binding protein (FKBP) (50). Similarly, rapamycin, another immunosuppressant synthesized by *Streptomyces hygroscopicus* (a macrolid originally described in 1975 as an antifungal agent), also binds FKBP and more likely the FKBP12 and FKBP52 isoforms (51, 52). The immunosuppressive effects of FK506 as well as of rapamycin are considered independent of the chaperone function of FKBP. When complexed with ligands, FKBP adopts a conformation allowing its binding to calcineurin and the mammalian target of rapamycin (mTOR). FKBP can also bind the inositol 1,4,5-triphosphate receptor (IP3R) Ca^2+^ channel, which is activated through phosphorylation by the protein kinase A (PKA), while its inactivation is induced through dephosphorylation by calcineurin (53, 54). FKBP also binds to the ryanodine receptor (RyR) channel and the type 1 transforming growth factor beta (TGFβ) receptor (55). Both CsA, FK506 (also known as fujimycin or tacrolimus) and rapamycin (or sirolimus) inhibit the phosphatase activity of calcineurin, thereby preventing the dephosphorylation of the nuclear factor of activated T cells (NF-AT). NF-AT is usually induced after Ca^2+^ binds to calmodulin, leading to the binding of calmodulin to calcineurin, a calcium–calmodulin-activated serine/threonine-specific phosphatase, which in turn is activated (52). In a model of liver fibrosis in rats, rapamycin was reported to inhibit mTOR, to demonstrate potent antifibrotic activity, and to improve portal pressure (56).

## Function of Cyclophilins

The main function of peptidylproline cis-trans isomerase, PPIases, is that of chaperone proteins involved in folding, assembly, and trafficking of other proteins (57, 58). The human genome encodes 17 cyclophilins: the peptidyl-prolyl isomerase A (PPIA or CyPA also called Cyp-18a a cytosolic protein of molecular mass 18 kDa) encoded by a gene located on chromosome 7, PPIB (CypB also called Cyp-22/p, an endoplasmic reticulum and Golgi protein of molecular mass 22 kDa) encoded by a gene on chromosome 15, PPIC (CypC an endoplasmic reticulum and Golgi protein of molecular mass 33 kDa), PPID (CypD a mitochondrial protein of molecular mass 20 kDa; the cytosolic CyPD and CyPF are named CyP40), PPIE (CypE, a component of the spliceosomal apparatus), PPIF (CypF is a component of the mitochondrial permeability transition pore involved in apoptosis regulation), PPIG (CypG or SR–cyclophilin or matrix–cyclophilin a nuclear matrix protein which interacts with RNA polymerase II is a component of the spliceosomal apparatus), PPIH (CypH), NKTR (Cypp), PPIL1 (encoded by the X-chromosome), PPIL2, PPIL3, PPIL4, PPIL6, PPWD1, RANBP2, and SDCCAG-10, respectively (59, 60). The CyPA exhibits multiple functions including folding of the procollagen I and transferrin, nuclear translocation of ERK1/2 kinases, transport of molecules to the plasma membrane through interaction with the Ig-like CD147 receptor, cholesterol transport, nuclear export of zinc-finger protein-1, and stimulation of apoptosis (61, 62). Although CyPA is mainly a cytosolic protein, there is also a secreted form of this molecule which is produced in response to different inflammatory stimuli, particularly infection (63). The secretion of CyPA is mediated *via* a vesicular transport pathway that depends on the Rho-kinase activation (64). The secreted form of CyPA acts as a chemoattractant for monocytes and leukocytes (63, 65, 66). To date, although several functions of most cyclophilin isoforms remain unknown, the different isoforms of cyclophilins exhibit domain-specific properties apart from their function as chaperones. For example, PPIA was found to bind the non-receptor tyrosine kinase Itk, playing a role in the maturation of thymocytes; PPIH and PPIL1, respectively, interact with the hPRP4 and SKIP proteins in the spliceosome, and PPIE shows a RNA-specific isomerase activity. Besides encoding 17 cyclophilins, the human genome encodes 18 FKBPs and three parvulins, the smallest PPIases (67).

**Figure 1:**
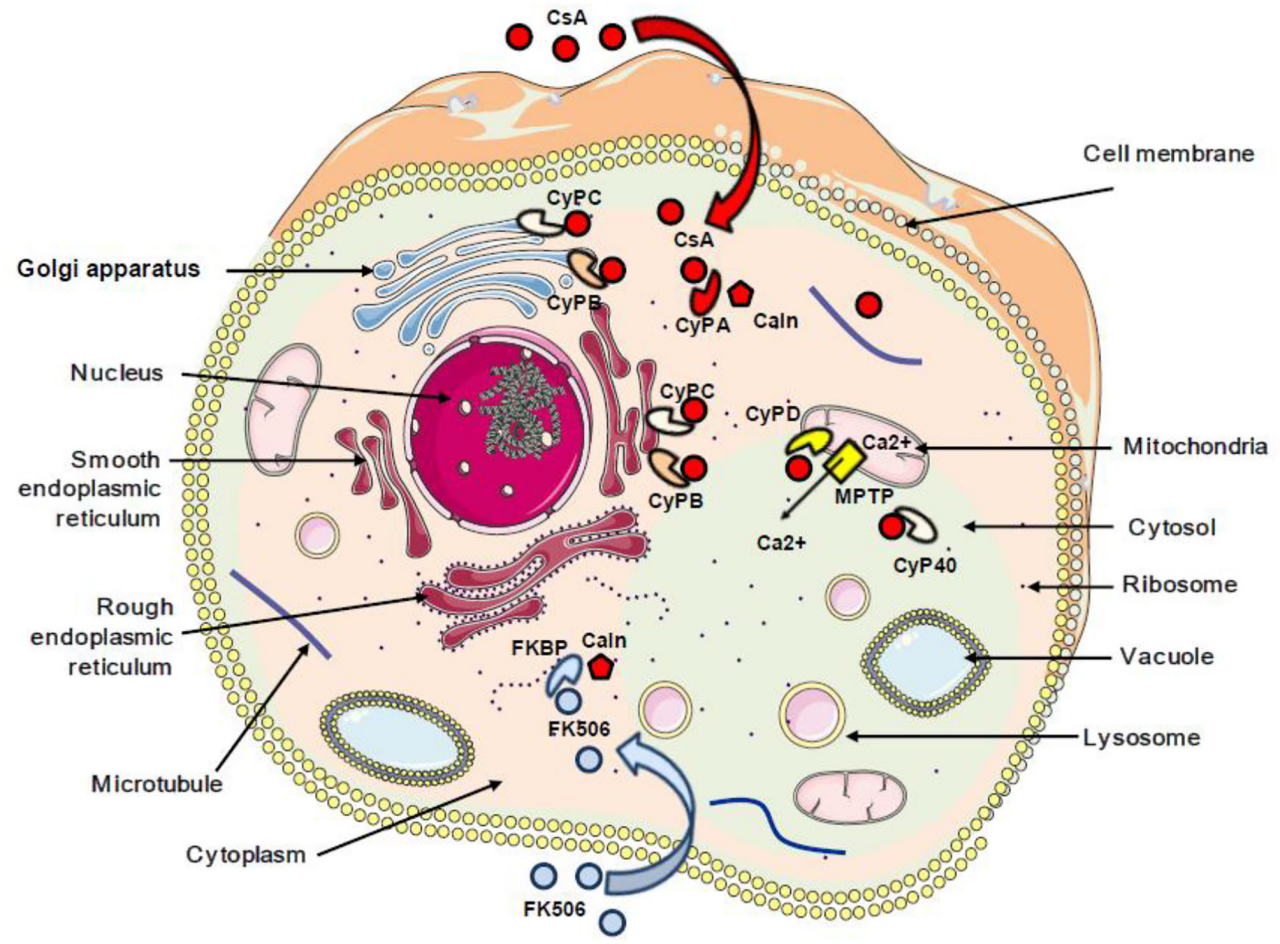
Schematic representation of the subcellular localization of cyclophilins and FKBP proteins. The red arrow indicates the interaction between cyclosporin A and cyclophilins. The blue arrow indicates the interaction between FK506 and FKBP. CsA, cyclosporin A; CyPA, CyPB, CyPC, CyPD, and CyP40, cyclophilins A, B, C, D, and 40; FKBP, FK506-binding protein; Caln, calcineurin; MPTP, mitochondrial permeability transition pore; Ca^2+^, calcium.

It was reported that CsA can bind PPIA, PPIB, PPIC, PPID, PPIE, PPIF, PPIG, PPIH, PPIL1, NKTR, and PPWD1, while PPIL2, PPIL6, RANBP2, and SDCCAG-10 are incompetent to ligate CsA (60). Special attention was given to the CsA/CypA interaction and a quantitative transcriptomics analysis (RNA-Seq) was performed to determine the tissue-specific expression of the CypA gene. This study indicated that CypA is ubiquitously expressed (68) (Figure 1).

## CsA Repurposing in AIDS Therapy: a Precedent in the Treatment of a Viral Disease With CsA

**Figure 2:**
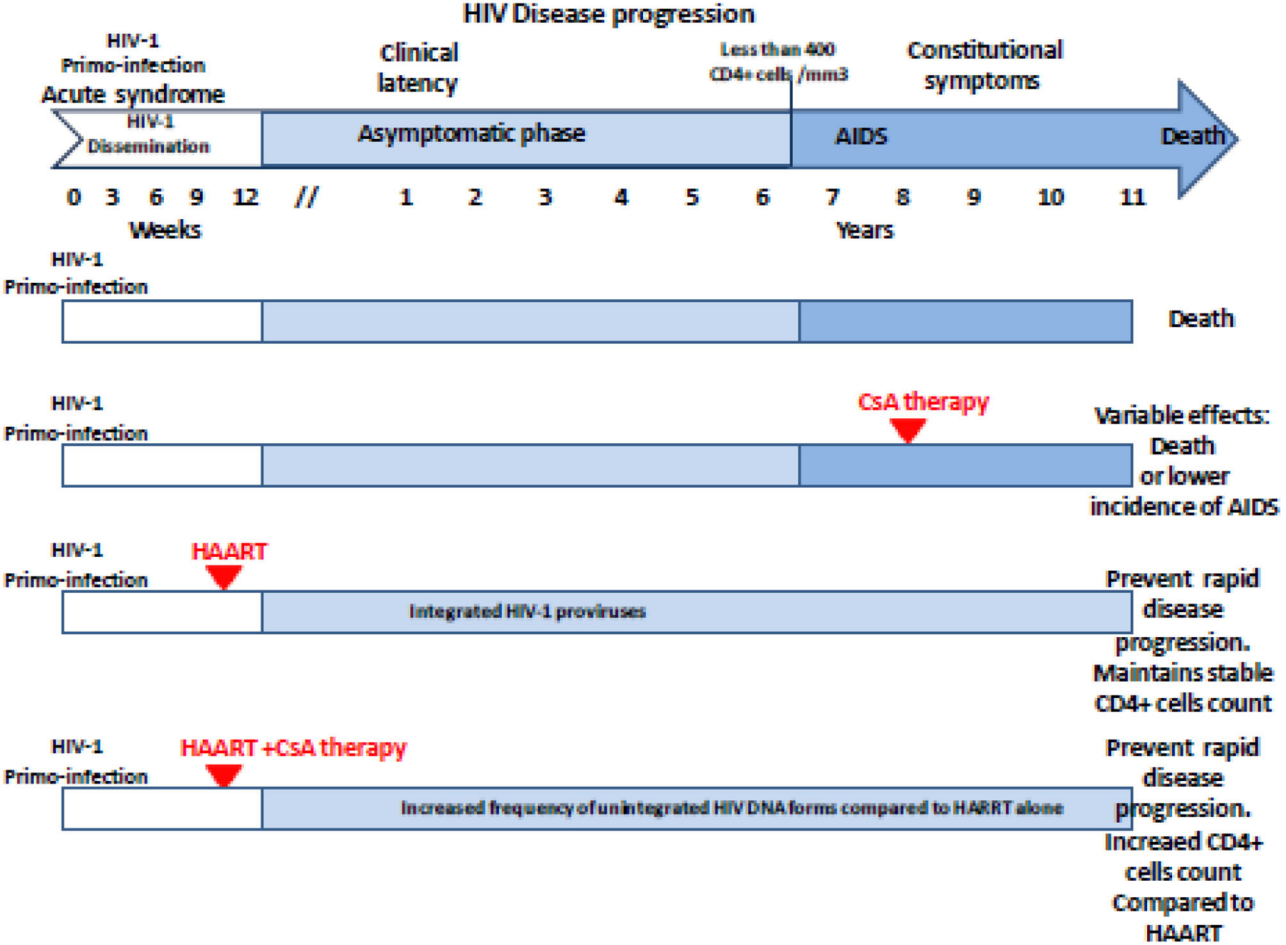
Schematic representation of the antiviral effect of CsA treatment on the HIV-1 disease progression regarding the clinical trials reported in the literature. The effectiveness and beneficial effects of CsA depend on the stage of the disease at which the treatment is given. Unintegrated DNA forms of viral genome increased in the CsA-treated group compared with controls when CsA is given post-primo-infection in association with HAART. AIDS, acquired immunodeficiency syndrome; HAART, highly active antiretroviral therapy; CsA, cyclosporine A.

Based on the hypothesis according to which the multiplication of the human immunodeficiency virus type 1 (HIV-1) in the organism is all the more important as the CD4 cells are activated, 25 years ago, CsA was considered as a possible drug to treat AIDS. During a press conference, the results of a preliminary CsA clinical trial carried out on AIDS patients by a team of medical doctors from the Laënnec Hospital (Paris, France) in October 1985 were reported (69). Unfortunately, after the death of two HIV patients under CsA therapy, a campaign fueled by media tended to discredit this work (70, 71). Among the criticisms that had been expressed, it was emphasized that using an immunosuppressant to treat a disease characterized by an immunosuppression (e.g., HIV-1-induced progressive depleted of CD4^+^ lymphocytes being at the origin of AIDS) was surprising. Despite the media attacks, the pilot phase was continued by the team of Andrieu who reported on the CsA treatment of eight patients who were given 7.5 mg CsA/kg daily and concluded, based on their observation, that clinical trials with CsA would be worth pursuing (72). However, adverse effects of this experimental treatment were reported by another team, which published the results of a CsA pilot study on nine patients with AIDS (six presented with *Pneumocystis carinii* pneumonia and three had Kaposi's sarcoma) who experienced severe toxic symptoms: one developed massive intravascular hemolysis and was withdrawn from the study after 13 days of treatment, the other also experienced severe symptoms which necessitated discontinuation of CsA therapy in six of them, and the condition of all patients improved after therapy was stopped (73). Although the results from these last clinical studies were disappointing, another study that enrolled 53 patients with renal transplantation, the HIV infection of whom was caused by an infected transplant or by blood transfusion, indicated that after 5 years the cumulative incidence of AIDS was lower in 40 patients who received CsA than in 13 transplant patients receiving immunosuppressive treatment without CsA (74). Several other reports highlighted a possible positive impact of CsA treatment on the progression of AIDS (75–79). Coming back to the animal model to explore pathophysiology without putting patients at risk, it was shown by the team of Fauci that administration of CsA to monkeys inoculated with the simian immunodeficiency virus (SIV) was beneficial relatively to the kinetics of CD4 cell depletion (80). This result revived the scientific debate on the use of CsA in the treatment of AIDS, but rather than using it as monotherapy on patients with declared AIDS (low CD4^+^ cell count), the choice fell on the use of CsA in combination with highly active antiretroviral therapy (HAART) during primary infection. This new therapeutic strategy was based on the hypothesis that rapid shutdown of T-cell activation in the early phase of primary infection could have long-term beneficial effect on the outcome of the disease. The team of Pantaleo reported that during a 64-week follow-up, patients receiving CsA in combination with HAART consistently maintained significantly higher levels of CD4^+^ T cells than those taking HAART alone (81). This promising result relaunched the investigation on the use of CsA in AIDS (27, 82–86) (Table 2). In 2014, De Iaco and Luban reported that CypA binds HIV-1 capsid (CA) and influences early steps in the HIV-1 replication cycle and that disruption of CypA binding to CA by CsA reduces the efficiency of HIV-1 transduction in some cells but not in others (90). More recently, Nicolas and colleagues reported the results of a clinical investigation, which concluded that unintegrated DNA forms of viral genome increased in the CsA-treated group compared with controls, suggesting an anti-integration effect of the drug (89) (Figure 2). This is consistent with earlier data demonstrating that cell activation is dispensable for viral entry but is required for the HIV-1 provirus integration (91–93). It will therefore have taken more than 30 years of research to begin to understand in which specific therapeutic conditions CsA can be beneficial in the treatment of AIDS. Finally, it was recently reported that CsA decreases HIV-1 infectivity by blocking CypA interaction with HIV-1 CA protein and incorporation of HIV-1 envelope glycoproteins (gp120 and gp41) into virions thereby impairing fusion with target cells (26). Altogether, these results suggest that treatment with CsA can be beneficial in the prevention of AIDS but that the window of action of this treatment is narrow, limited to primary infection to prevent the integration of the viral genome, while it is no longer efficient on chronic infection once the provirus is already integrated into the DNA of infected cells.

**Table 2:** *In vitro* effect of CsA on HIV replication and on disease progression in HIV-infected patients.

**Date**	**Type of study**	**Results**	**References**
***In vitro***
1988	HIV *in vitro* infection and replication H-9 T-cell leukemic line human peripheral blood-derived lymphocytes	Pretreatment of cells and human lymphocytes with CsA over 24 h prevented viral infection over a 21-day period, whereas the addition of drug at 2 h postinfection with HIV-1 had a significant inhibitory effect on viral replication and expression of the virus-specific antigens p17 and p24^gag^	(87)
1992	HIV and CD4 T cells	CsA induced a 100-fold reduction in the yield of HIV infection CsA inhibited the growth of HIV infected cells	(75)
1994	HIV T4 lymphoid cell lines, in a monocytic cell line, and in HeLa T4 cells	SDZ NIM 811 selectively inhibited HIV-1 replication in CD4+ lymphoid cell lines, in a monocytic cell line, and in HeLa T4 cells	(88)
2010	HIV and Human CD4+-T cells	CsA inhibited HIV infectivity	(26)
2013	HIV and T cell line or peripheral blood mononuclear cells	CsA inhibited HIV-1 replication in a GFP indicator T cell line and peripheral blood mononuclear cells	(27)
**In patients**
1978	Transplanted patients (*n* = 7)	CsA was effective in inhibiting rejection (adverse effect: nephrotoxicity and hepatotoxicity.)	(41)
1988	AIDS patients (*n* = 8)	CsA (7.5 mg/kg daily) Sustained and increased > 600 CD4+ cells/mm3, decreased CD8+ cell count. Lymphadenopathy disappeared. Reversibility once CsA was stopped	(72)
1989	AIDS patients (*n* = 8)	Severe toxic syndrome requiring discontinuation of CsA Decreased lymphocyte count, CD4+ and CD8+ T- cells, and no resolution of symptoms	(73)
1993	Transplanted kidney patients & HIV-1 (*n* = 53)	5-year cumulative risk of AIDS: 31% in CsA group vs. 90% in non CsA group, *P* = 0.001	(74)
2002	9 early HIV patients treated HAART + CsA	Significantly higher CD4+ T cells in patients treated with CsA	(81)
2004	3 HIV patients treated HAART + CsA	Pharmacological adjustment of CsA in association with HAART	(83)
2010	54 early HIV (ART + CsA vs. ART)	No apparent immunological and virological benefit	(86)
2017	20 early HIV (ART + CsA vs. ART)	Increased non-integrated DNA in the CsA arm between weeks 0 and 36 weeks CsA has unintegrated effect	(89)

## Is There a Perspective for CsA Repurposing in COVID-19?

Immunocompromised patients including patients with HIV, those receiving immunomodulatory therapy for autoimmune disease, patients with cancer, and solid organ transplant recipients who are immunosuppressed to prevent complication associated to alloimmune responses are generally considered at risk for more severe viral infection because of their poor immune response. In transplant recipients, CsA and tacrolimus calcineurin inhibitors are the most prescribed drugs for the prevention of alloimmune responses (41, 94). Therefore, the question of using CsA in COVID-19 recently comes into debate since it remains unclear if immunosuppression in transplanted patients alters the predisposition to acquiring COVID-19 and/or modifies the disease outcome for better or worse (95). Today, solid organ transplant recipients are listed as high-risk individuals for the development of severe forms of COVID-19 (96), and there is a specific follow-up of transplanted patients to evaluate their outcome when they become infected with SARS-CoV-2. It is generally admitted that immunosuppressive therapy in transplanted patients modulates humoral and cell-mediated immunity increasing the risk of severe infection when exposed to viruses (97). In regard to this idea, some authors suggested pausing immunosuppressant drugs as a precaution in transplanted patients found positive for SARS-CoV-2 (98). Yet, it was also reported that transplanted patients have not been found more susceptible to viral infections and severe forms of COVID-19 than the general population (99–101), which begs questions about the relationship between CsA treatment and COVID-19. An observational clinical study from Spain which followed 29 kidney transplant recipients with COVID-19 reported a mortality of 12.5% in the group of patients under CsA therapy (*n* = 23) compared with 50% mortality in the control group with reduced doses in CsA (*n* = 6), supporting the hypothesis that CsA therapy is safe and might be beneficial to transplanted patients with COVID-19 (102). However, this study should be interpreted with caution due to variability of other drugs used in these patients. Observational studies have shown that patients receiving CsA for the prevention of graft vs. host (GVH) disease have a lower risk of developing a COVID-19 infection than patients receiving basic treatment with tacrolimus or corticosteroids (Table 3). Interestingly, in a recent study including 40 kidney-transplanted patients, Demir and colleagues identified by using a multivariable analysis that the use of CsA was associated with a lower incidence of death [0.077 (95% CI, 0.018–0.324; *P* ≤ 0.001)] (105). The question currently being raised is whether the background immunosuppressive therapy in transplanted patients should be modified, when possible, by CsA to prevent the occurrence of COVID-19 (100).

**Table 3:** Cyclosporin A based treatment in transplanted patients.

**No. of transplanted patients**	**Cysclosporin A**	**Corticoids**	**Intensive care unit (ICU)**	**Death**	**References**
**Heart**					
6 transplanted patients	6/6 patients received Cysclosporin A (70–200 mg/d)	NA	2/6 patients admitted inICU (2 and 16 days)	2 died: 1 with acute respiratory distress syndrome. 1 with sepsis. Their Cysclosporin A therapy was reduced in both cases (100 and 40%, respectively)	(103)
**Kidney transplantation**					
2 patients	1 patients	NA	1 patient not treated with Cysclosporin A	1 patient not treated with Cysclosporin A	(104)
40 patients	5 patients (12%)	40 (100%)	SEVERITY Cysclosporin A associated reduction risk of mortality multivariate analysis OR: 0.077 (IC0, 018–0.32) *p* <0.001		(105)
19/2,493 kidney transplant recipient	9/19 patients (47.4%)	NA	NA	2 patients (22%) died in the cyclosporin A treated group vs. 7 patients alive (70%) *p* = 0.03	(106)
23 patients	6 patients already treated with Cysclosporin A 19 patients switched to Cysclosporin A therapy	NA	NA	Mortality was higher in the immunosuppression minimization strategy group, 3/6 patients (50%), as compared to the Cysclosporin A strategy group 3/23 patients (13%)	(102)
**Liver transplantation**					
151 reports SARS CoV 2 with liver transplantation	8 patients	67 (44%)	NA	4/28 died patients received Cysclosporin A vs. 4/123 alive patients (non-significative)	(107)

At least eight FDA-approved clinical trials of CsA and FK506 are currently underway in patients with severe COVID-19 (Table 4). The majority of the clinical trials presented in Table 4 are still ongoing and no results have been disclosed. Preliminary results (not certified by peer review) made available recently indicate that CsA (9 mg/kg/day) in short courses of treatment for COVID-19 patients requiring oxygen (clinical trials NCT04412785; first posted February 6, 2020) is safe and associated with significant reductions of hyperinflammation (108). An open-label, non-randomized pilot clinical study on 209 adult patients confirmed positive for SARS-CoV-2 receiving enoxaparin, methylprednisolone, or prednisone compared the clinical outcome of 105 patients who received CsA (oral CsA at a dose of 1–2 mg/kg daily) plus steroids to that of 104 patients treated with steroids alone; this study concluded that CsA used as adjuvant to steroid treatment improves the outcomes of patients with moderate to severe forms of COVID-19 and reduces mortality (109).

**Table 4:** FDA approved clinical trial proposing cyclosporine A to treat SARS-CoV-2 infection.

	**Clinical trial**	**Study title**	**Intervention**	**Countries**
1	NCT04412785	Cysclosporin in Patients With Moderate COVID-19	Phase 1 safety study to determine the tolerability, clinical effects, and changes in laboratory parameters of short course oral or IV Cysclosporin (CSA) administration in patients with COVID-19 disease requiring oxygen supplementation but not requiring ventilator support.	University of Pennsylvania Philadelphia, Pennsylvania, United States
2	NCT04392531	Clinical Trial to Assess Efficacy of cYclosporine Plus Standard of Care in Hospitalized Patients With COVID19	Open, Controlled, Randomized Clinical Trial to Evaluate the Efficacy and Safety of Cysclosporin Plus Standard Treatment vs. Standard Treatment Only in Hospitalized Patients With COVID-19 Infection	Complejo Hospitalario Universitario La Coruña La Coruña, Galicia, Spain Hospital Quiron La Coruña La Coruña, Galicia, Spain Hospital Rey Juan Carlos Mostoles, Madrid, Spain
3	NCT04540926	Cysclosporin A Plus Low-steroid Treatment in COVID-19 Pneumonia	Consecutive patients with suspected or confirmed diagnosis of COVID-19 were assigned, in an unblinded and non-randomized fashion, to receive either steroids plus CsA (intervention group) or steroids only (standard of treatment in this hospital, control group), as per individual clinical judgment	Jose Luis Jl Galvez-Romero Puebla, Mexico
4	NCT04492891	Cysclosporin For The Treatment Of COVID-19(+)	Phase IIa clinical trial in which 75 non-ICU hospital inpatients will be randomized 2:1 to 7 days of Neoral (2.5 mg/kg PO BID) + standard of care (SOC) or no CSA + SOC.	Baylor College of Medicine Houston, Texas, United States
5	NCT04451239	Topical Steroids and Cyclosporin-A for COVID-19 Keratoconjunctivitis	Single Group Assignment All patient will be treated with Topical 1% prednisolone acetate for 7 days as initial treatment + non-preserved artificial tears and Cysclosporin A 0.5% four times daily.	Farawanyia hospital Kuwait, Farawanyia, Kuwait
6	NCT04341038	Clinical Trial to Evaluate Methylprednisolone Pulses and Tacrolimus in Patients With COVID-19 Lung Injury	Open Randomized Single Centre Clinical Trial to Evaluate Methylprednisolone Pulses and Tacrolimus in Patients With Severe Lung Injury Secondary to COVID-19	Hospital Universitari de Bellvitge L'Hospitalet de Llobregat, Barcelona, Spain
7	NCT04420364	Maintenance vs. Reduction of Immunosuppression for Renal Transplant Patients Hospitalized With COVID-19 Disease	Maintenance or reduction of immunosuppression, phase II-III Single-blind, parallel-group, randomized, active-controlled trial	Birgham and Women's Hospital, Boston, Massachusetts
8	NCT04569851	Clinical Characteristics and Prognostic Factors of Patients With COVID-19 (Coronavirus Disease 2019)	Retrospective, observationnal Clinical Characteristics and Prognostic Factors of Patients With COVID-19 Using Big Data and Artificial Intelligence Techniques (BigCoviData)	Hospital Universitario de Guadalajara Guadalajara, Spain Hospital Universitario La Princesa Madrid, Spain

Altogether, these results suggest that CsA could have a beneficial effect in the treatment of COVID-19 patients and that such repurposing strategy should be further investigated while being aware of possible side effects. In addition, these data also raise questions about the mechanisms by which CsA might influence the outcome of COVID-19.

## CsA and Cyclophilin in Proinflammation Processes: Implication for COVID-19

Upon entering the cell, the immunosuppressants CsA and FK506 bind with high affinity to CyPs (also named immunophilins) and inhibit their peptidyl prolyl cis-trans isomerase activities. The CyP–CsA (or FKP–FK506) complex binds to calcineurin and inhibits its phosphatase activity. Many of the suppressive actions of CsA on T cells appear to be due to an inhibition of T-cell receptor (TCR)-induced activation signals with minimal effects on already activated CD8^+^ cytotoxic T cells (110). Although CSA affects T-cell differentiation and proliferation and cytokine production, these cells still express the interleukin-2 receptor (IL-2R) and proliferate under IL-2 stimulation (111, 112). However, CsA can apparently also trigger a status on T-cell-mediated autoimmunity (113). CsA inhibits the development of both CD4^+^CD8^neg^ T-cell and CD4^neg^CD8^+^ T-cell lineages (114). CsA inhibits a T-cell receptor-dependent and calcium-dependent signal transduction pathway and blocks T-cell proliferation by inhibition of the IL-2 synthesis, and this is achieved after forming a complex with CyPA. In the absence of CsA, TCR-induced activation signal triggers Ca^2+^ binding to calmodulin that leads calmodulin to form a complex with calcineurin, a calcium/calmodulin-dependent serine threonine phosphatase. The activation of calcineurin triggers dephosphorylation of the cytoplasmic nuclear factor of activated T cells (NF-ATcP). Once dephosphorylated, NF-ATc translocates from the cell cytoplasm into the cell nucleus and activates the transcription of the IL-2 gene (115). Under CsA treatment, the CsA/CyPA complex specifically binds to calcineurin and inhibits its phosphatase function (116, 117). Due to a lack of phosphatase activity, the nuclear factor of activated T cells (NF-AT) remain under its inactive cytoplasmic phosphorylated form (NF-ATcP). *In vivo* studies have highlighted that CsA promotes the expansion of Foxp3^+^ T regulator cells (Treg) (118). Indeed, the result of CsA treatment is a change in the balance between T helper cells and Treg cells that favor the Treg population. The CypA is regulated by inflammatory stimuli, and several cell types secrete CypA in response to oxidative stress. Zhang and colleagues also reported that serum CypA concentration correlates with serum interleukin-6 (IL-6), matrix metalloproteinase-9 (MMP-9), and C-reactive protein expression (119). It was recently reported that the secreted CypA can be used as a potential inflammatory biomarker of chronic obstructive pulmonary disease (COPD), as its expression levels are elevated in serum of COPD patients and reflects the severity of inflammation (119).

## Pathological Similarities Between Transplanted Patients and COVID-19 Patients: Tissues Injured With Picture of Chronic Vascular Rejection

**Figure 3:**
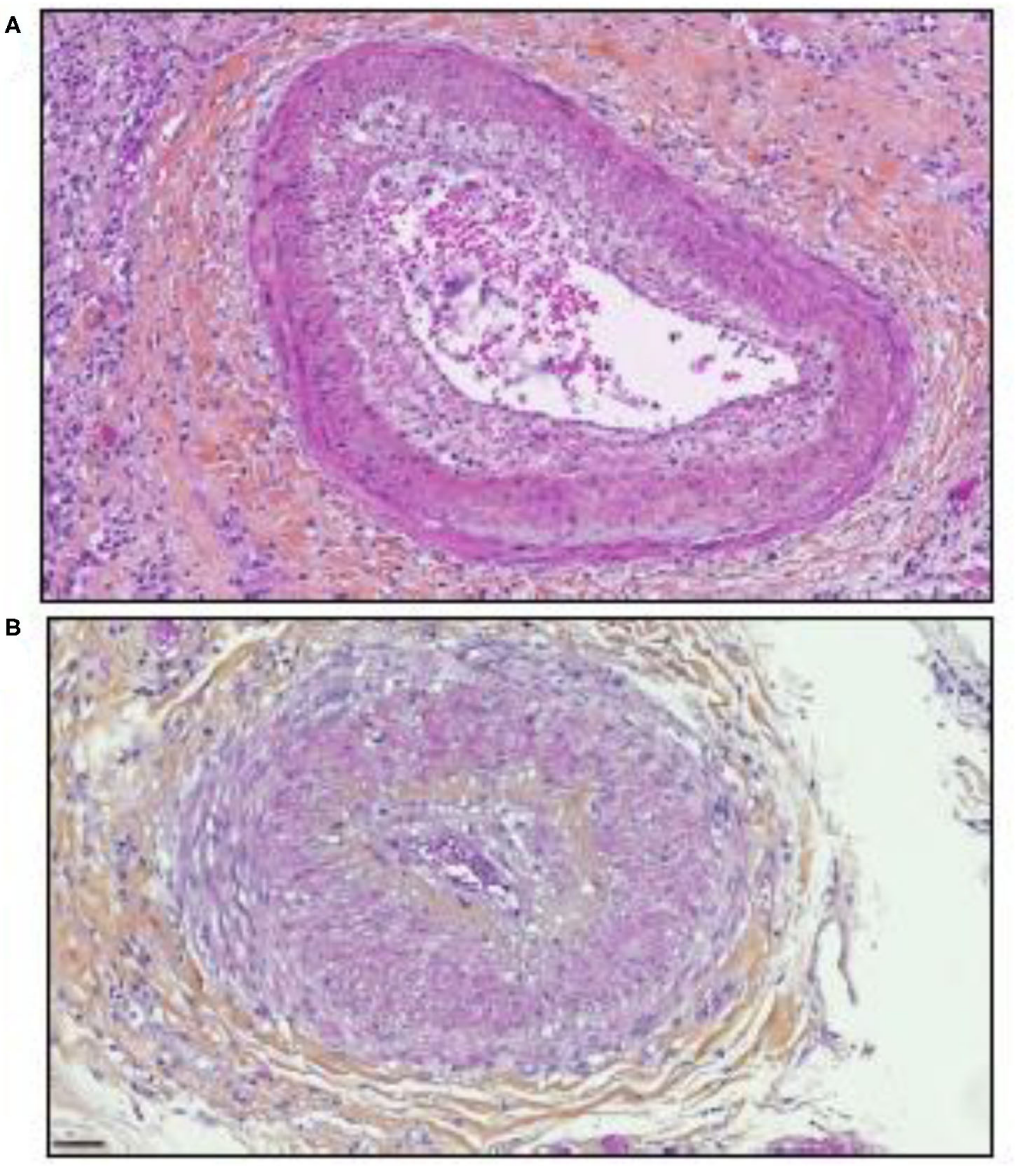
Illustration of the microscopic examination of histological sections of tissues from patients who died of COVID-19 (postmortem formalin lung sample from medical autopsy performed in the forensic medicine department of Marseille Hospital). The histological sections were stained using hematoxylin, eosin, and saffron (hematoxylin stains the cell nuclei blue, eosin stains the extracellular matrix and cytoplasm pink, the saffron stain in orange the conjunctive matrix). **(A)** Vascular rejection is characterized by concentric thickened artery secondary to intimal proliferation and endovasculitis. Original magnification × 150. **(B)** Concentric thickened artery secondary to fibrointimal proliferation. Original magnification × 200 μm.

Significant parallels are observed between SARS-CoV-2 tissue injury (120, 121) and allograft rejection and especially with chronic vascular rejection (122, 123). In tissues of patients who died from COVID-19, similar lesions to those observed in chronic vascular rejection grade D were observed (122). Vascular rejection is characterized by concentric thickened arteries and/or veins, due to fibrointimal connective tissue. These lesions usually start with intimal proliferation, then fragmented and discontinuous internal elastic lamina (120, 121), as illustrated in Figure 3. Concurrent endovasculitis has also been observed (123). In patients suffering from GVH disease, lung histological lesions are characterized by alveolar changes (intra-alveolar fibrin, organizing pneumonia, and chronic interstitial pneumonia), atypical pneumocytes, intra-epithelial bronchiolar T cells, and perivenular cuffing (124–127).

**Figure 4:**
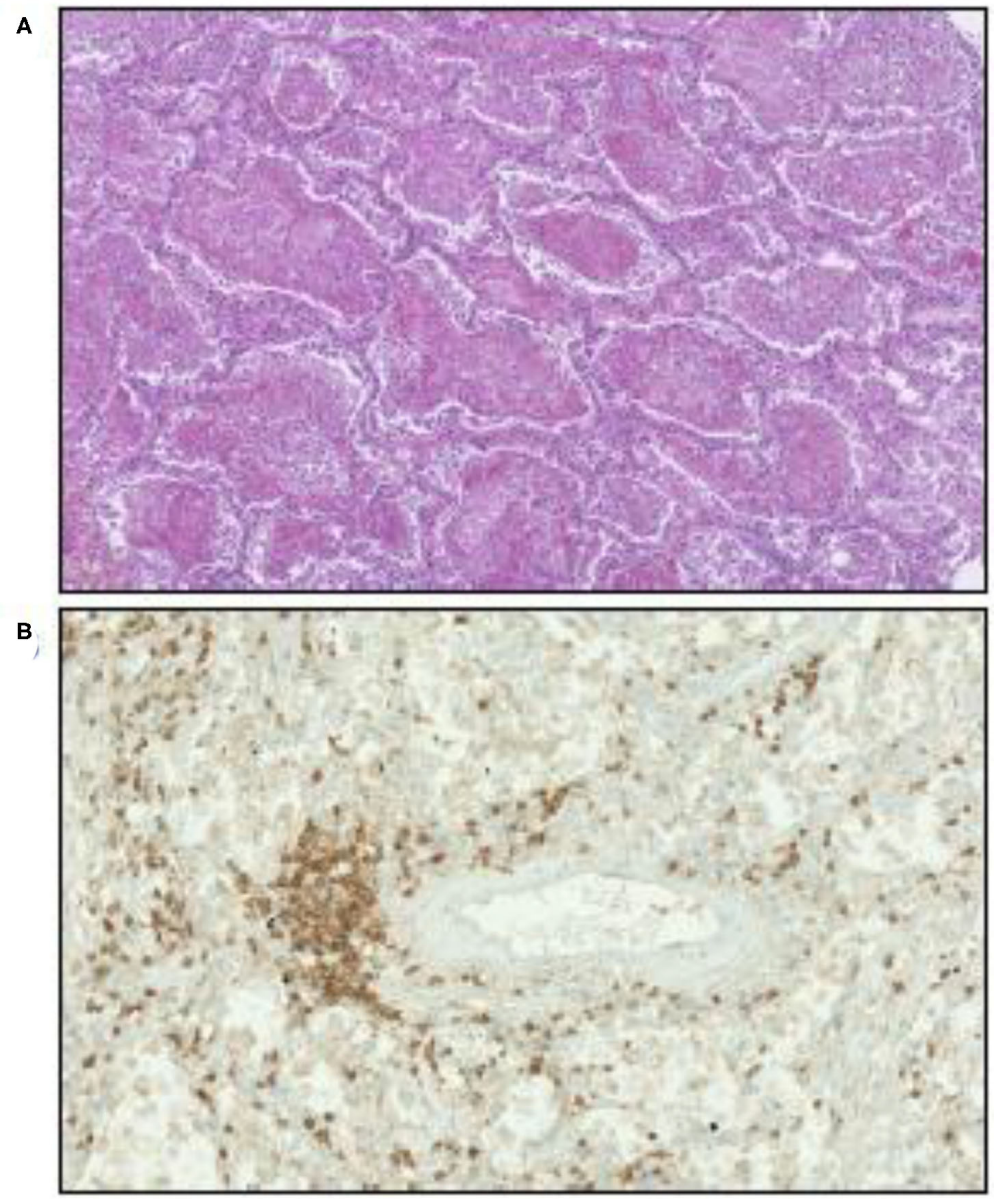
Illustration of microscopic examination of tissues from patients who died of COVID-19 (postmortem formalin lung sample from medical autopsy performed in the forensic medicine department of Marseille Hospital). **(A)** Hematoxylin, eosin, and saffron staining showing intra-alveolar fibrin. Original magnification × 70. **(B)** Inflammatory perivascular lymphocytes T infiltration evidenced by anti-CD3 monoclonal antibody immunostaining. Original magnification × 170.

Lung analysis of patients who died from COVID-19 showed an inflammatory perivascular lymphocyte infiltration (120, 121), as illustrated in Figure 4, that presents some similarities to those observed in GVH, although non-specific (128). Perivascular inflammation was reported to be patchy and scattered, composed mainly of lymphocytes, with thrombi in the branches of the pulmonary artery and focal areas of congestion in the alveolar septal capillaries, as well as septal capillary lesions with wall and luminal fibrin deposition (128).

In these diseases, critical epithelial stem cell populations are preferentially targeted: in one instance by cytotoxic immune pathways, in the other by a viral protein–receptor interaction. Moreover, in both diseases again, severe injuries are mediated by cytokine deregulation named the “cytokine storm syndrome” which leads to cell apoptosis. Cytokine dysregulation has historically been reported in the early phase of acute GVH disease described by Ferrara as a “cytokine storm” (129) and subsequently used to describe the exacerbated immune response observed in severe COVID-19 infection (130, 131). Thus, it could explain some of the histological similarities observed, even chronic, since physiological mechanisms involved in these lesions are in part common. Stem cell death by apoptosis is associated with activation of the p53–p73 “suicide pathway” observed in GVH disease, and perivascular lymphocyte infiltrates were identified in case of GVH disease (132–135).

## COVID-19 Infection in Transplanted Patients

Recipients of allogeneic hematopoietic stem cell transplant (HSCT) are generally considered at particular risk of developing severe forms of COVID-19 when infected with SARS-CoV-2 due to the profound immunosuppression related to transplant-associated anti-rejection therapy expected to reduce the immune defense of the host thereby favoring *in vivo* viral replication. It was reported that treatment with the selective JAK1/2 inhibitor ruxolitinib has shown promising results in the context of COVID-19 patients with GVH disease (136). In COVID-19, tissue injury observed in patients with severe forms of the disease appears to be related to a massive increase of inflammatory cytokine level and increase of CD15^+^CD16^+^ neutrophils known for being involved in proinflammatory processes (137, 138). It is currently admitted that severe forms of COVID-19 are associated with a release of cytokines and chemokines such as IL-2, IL-6, IL-7, IL-10, tumor necrosis factor (TNF), and granulocyte colony-stimulating factor (GCSF) (2, 139).

**Figure 5:**
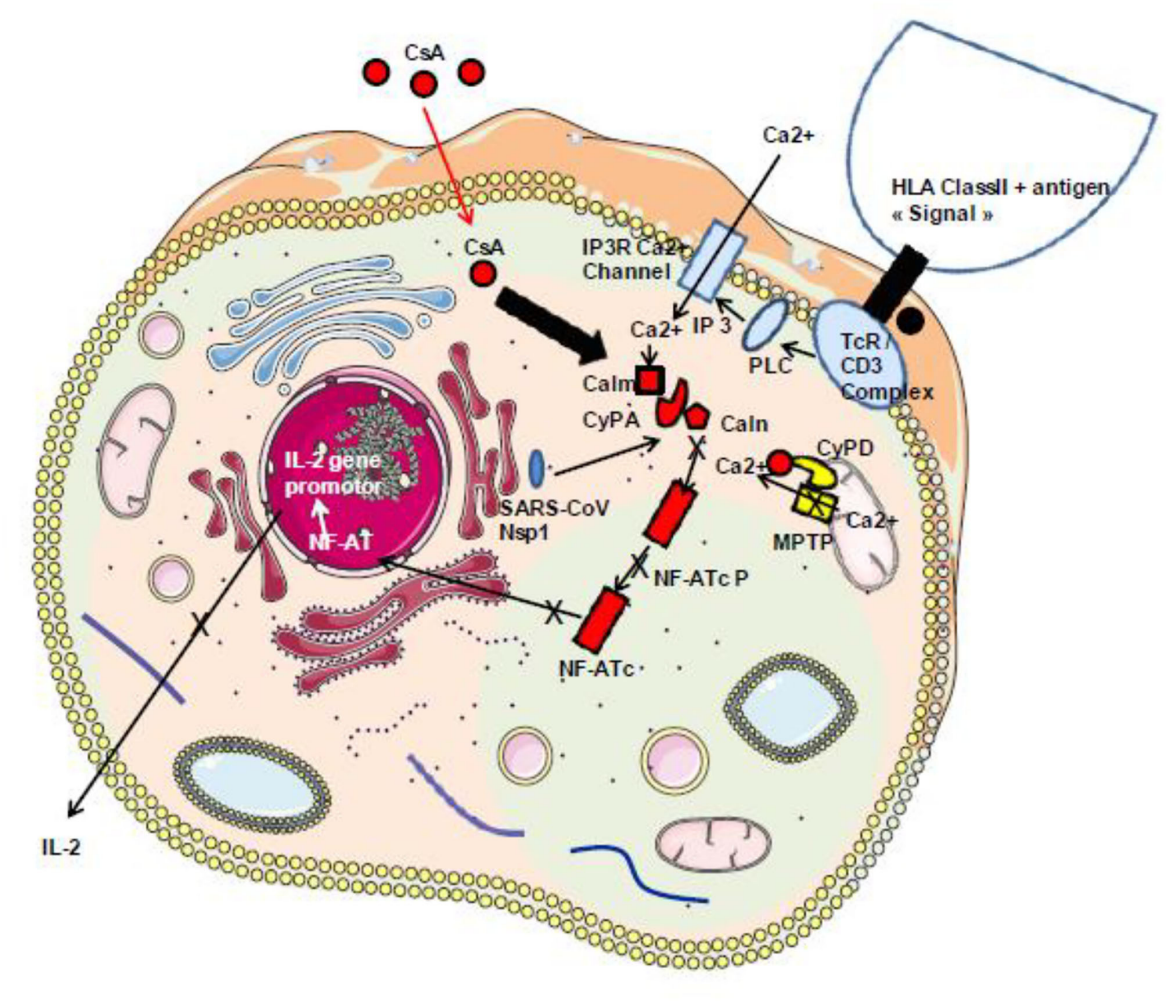
Schematic representation of the classical TcR/CD3-induced activation of IL-2 production. During infection with SARS-CoV-2, the virally induced cell dysregulation leads to the aberrant opening of MPTP inducing mitochondrial release of Ca^2+^ that triggers an abnormal Ca^2+^/calmodulin activation of calcineurin and dephosphorylation of the cytoplasmic nuclear factor of activated T cells (NF-AT) leading to NF-AT nuclear translocation and the synthesis of IL-2 and other inflammatory cytokines. Under CsA treatment, the CsA/CyPA complex specifically binds to calcineurin and inhibits its phosphatase function. Consequently, the NF-AT remain under its inactive cytoplasmic phosphorylated form. Moreover, by interacting with CyPD, CsA prevents the opening of MPTP and the release of Ca^2+^ that usually lead to cell death. In addition, through binding to CyPA, CsA is expected to upregulate interferon that blocks virus replication. HLA class II, human leukocyte antigen class II; TcR–CD3 complex, T-cell receptor–CD3 complex; PLC, phospholipase C; IP3, inositol 1,4,5-triphosphate; Calm, calmodulin; Caln, calcineurin; NF-ATcP, nuclear factor of activated T-cell cytoplasmic phosphorylated form; NF-ATc, NF-AT cytoplasmic dephosphorylated; PKC, protein kinase C; CsA, cyclosporin A.

Among these cytokines, therapeutic approaches targeting excessive inflammation caused by IL-6 interaction with its cellular receptor IL-6R have been under investigation using IL-6 antagonists such as tocilizumab and sarilumab used in the treatment of autoimmunity (140–143). It was recently shown that the total number of CD4^+^ T cells, CD8^+^ T cells, B cells, and NK cells in patients was markedly decreased in the most severe forms of COVID-19 and that there is an increase of IL-2, IL-6, IL-10, and IFN-γ (131, 144–146). There is likely space for investigating the possible beneficial effect of immunosuppressant CsA therapy in COVID-19, since this molecule is known to reduce IL-2 production that contributes to the cytokine storm reported in the severe forms of COVID-19 (Figure 5). It is also worth noting that the Nsp1 protein found to have multiple functions (e.g., binds to 40S ribosomal subunit and inhibits translation, triggers host mRNA degradation by endonucleolytic cleavage, induces cell cycle arrest, inhibits IFN signaling) was reported in SARS-CoV to enhance IL-2 production when overexpressed and that SARS-CoV infection increases signaling through the calcineurin/NF-AT (147). Such Nsp1 induction of IL-2 production is probably also occurring with SARS-CoV-2.

## CsA and Cyclophilin in Viral Infectious Processes: Implication for COVID-19

Different isoforms of cyclophilins CyPA and CypB were reported to specifically bind a proline-containing sequence in the polyprotein Pr55^gag^ and the p24^gag^ capsid protein of the HIV-1, and CsA disrupts the interaction of these proteins with CyPA and also with CyPB although with less efficiency (148). *In vitro*, CsA was reported to inhibit the replication of HIV-1 (149). The non-immunosuppressant analog of CsA, SDZ NIM 811 (Sandoz), was also found to inhibit HIV-1 *in vitro* (150).

Besides HIV-1, CsA was reported to inhibit the vesicular stomatitis virus (37), the hepatitis C virus (HCV) (151, 152), the human papillomavirus (HPV)-16 (153), the influenza A virus (154), and the Rift Valley fever virus (155). Regarding the HCV, the RNA-dependent RNA polymerase NS5B from the virus binds the human CypA and CypB proteins (156, 157), and CypA was also found to interact with the NS2 protein of HCV (158), while CypB appeared to regulate the HCV polymerase and CyP40 seemed to also be involved in HCV replication (159). First, a 3.5 log reduction of HCV load was demonstrated with the CsA analog DEBIO-025 (160). In light of these results, clinical trials of Cyp inhibitors (DEBIO-025, SCY635, and NIM811) have started against HCV, and a very elegant *in vitro* work evidenced that NIM811 reduces HCV replication by inhibiting CyPs, including CyPA, CypH, and CyPE, and identified many cellular compounds interacting with these CyPs (161).

Similarly, in flaviviruses, it was reported that CsA blocks the West Nile virus, dengue-2 virus, and yellow fever virus replication. CsA was found to inhibit the interaction between CypA and the NS5 protein (and also CyPA and viral RNA) of the West Nile virus (38), while CyPB was found to interact with the NS4A protein of the Japanese encephalitis virus (162), suggesting that CyP isoforms are essential to the replication complex of flaviviruses.

Regarding coronaviruses, it was reported that CsA inhibits the human coronaviruses HCoV-NL63, HCoV-229, and SARS-CoV-1, as well as animal coronaviruses such as feline CoV and porcine CoV, suggesting that CyPs are required for the successful replication of most coronaviruses (147). Once inside the cell, the genomic RNA (positive) from each coronavirus is released from the viral particle present in late endosomes. Covered with a cap allowing its anchorage to the ribosome level, this genomic RNA serves as a template for the translation of two large open reading frames (ORF1a and ORF1b). This yields to the synthesis of the polyprotein 1a (pp1a), and following a ribosomal frameshift, it leads to the extended pp1ab polyprotein. After proteolysis, several non-structural proteins (Nsp) are produced including a RNA-dependent RNA polymerase which interacts with other Nsp compounds to form, together with the host protein including CyP proteins, the endoplasmic-reticulum-derived double-membrane-associated replication transcription complex required for the synthesis of all viral molecules which enter in the composition of *de novo* viral particles (163–165). The antiviral properties of CsA against HCoV-229E and SARS-CoV-1 were confirmed in an independent *in vitro* work which concluded that CsA strongly affects the replication of coronaviruses HCoV-229E and SARS-CoV-1 rendering RNA and protein synthesis almost undetectable (19). It was also reported that CyPA interacts with the SARS-CoV-1 nucleocapsid (N) protein (166, 167). A genome-wide SARS-CoV-1 screening of viral proteins interacting with cellular compounds (human cDNA libraries) performed using the yeast two-hybrid strategy revealed that the Nsp1 protein of SARS-CoV-1 binds FKBPs (147). It was also reported that FK506 inhibits the replication of HCoV-NL63, HCoV-229, and SARS-CoV-1 and that inhibition of HCoV-NL63 replication by FK506 occurs through inhibition of the FKBP1A/B, suggesting that both FKBP and CyP families of PPIases are involved in the replication of coronaviruses (24). It is worth noting that both siRNA-mediated CyPA depletion and shRNA-mediated CyPA depletion so far failed to trigger the reduction of SARS-CoV-1 replication, suggesting either that SARS-CoV-1 transcription mainly involves FKBPs and/or CyP other than CyPA or that the residual CyPA present in cells after treatment was sufficient to achieve the building of the replication complex (19, 168). CsA was also reported to inhibit the replication of MERS-CoV, a result which was more drastic when CsA was combined with interferon (IFN)-α (169). It was reported that CsA upregulates the interferon regulatory factor 1 (IRF1) signaling pathway and that inhibition of IRF1 allows viral replication despite the presence of CsA. The SARS-CoV-1 virulence factor Nsp1 antagonizes the IFN immune response (170, 171).

**Figure 6:**
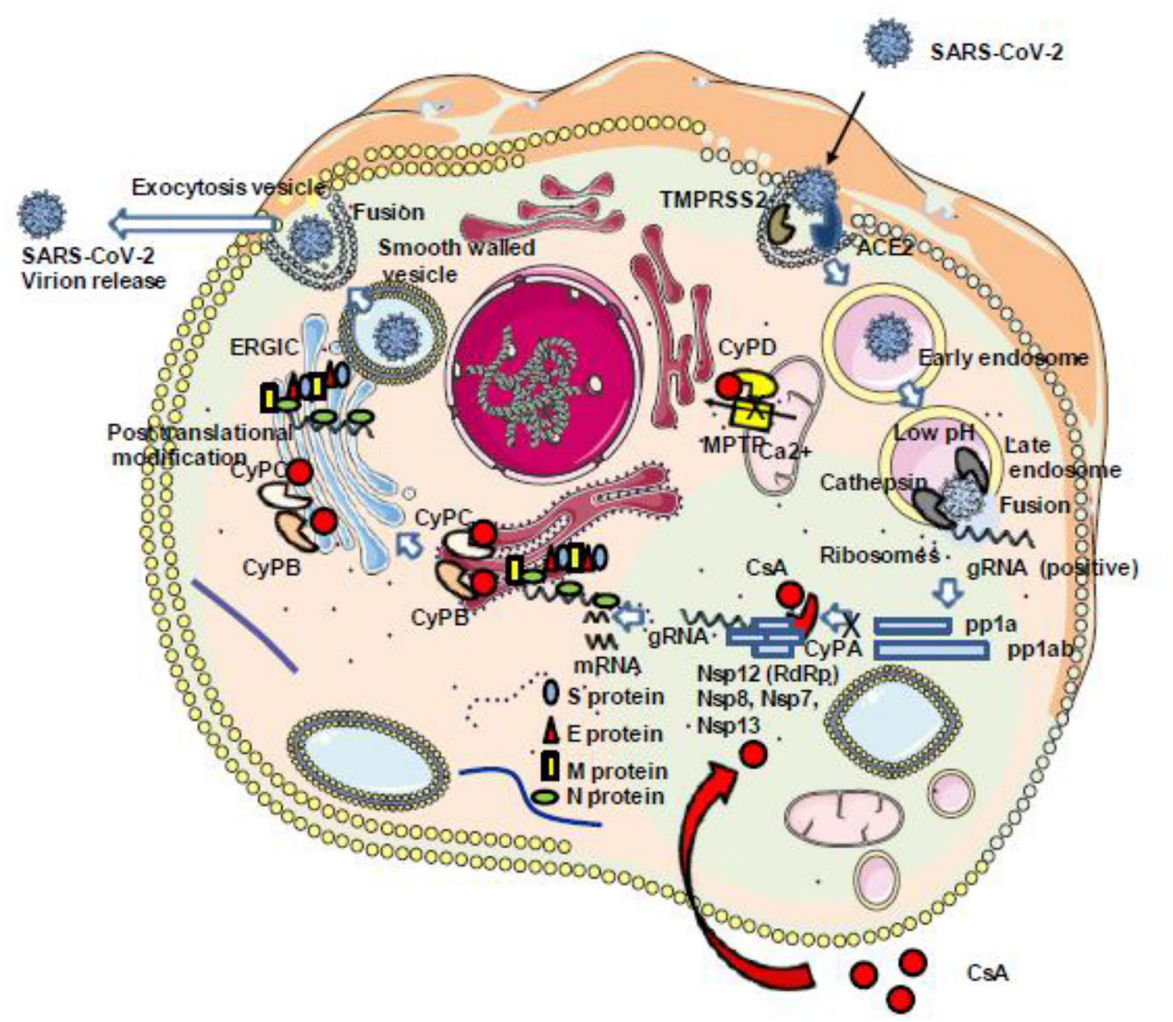
Schematic representation of the antiviral properties of CsA. Once the SARS-CoV-2 genome starts to be transcribed into pp1a and pp1ab, the RNA-dependent RNA polymerase (Nsp12) should interact with several other viral (Nsp8, Nsp7, Nsp13) and cellular (CypA) proteins to construct a replication complex. This complex is required for the viral replication cycle to be completed with the synthesis of the structural proteins S, E, M, and N. This step can be inhibited through the interaction between CsA and CypA (see text for details regarding the different steps of the SARS-CoV-2 cycle which can be inhibited by CsA). ACE2, angiotensin-converting enzyme 2; CsA, cyclosporin A; CyPA, CyPB, CyPC, and CyP, cyclophilins A, B, C, and D; gRNA, genomic RNA; Nsps, non-structural proteins; ERGIC, endoplasmic reticulum–Golgi intermediate compartment.

During the replication cycle of SARS-CoV-2, the RNA-dependent RNA polymerase (RdRp) required for the replication of the virus is active within a complex composed by several non-structural proteins of the virus such as Nsp12, Nsp8, and Nsp7 as well as cellular proteins likely including members of the CyP protein family. Within this replicative machinery (that is a target for the FDA-approved triphosphate metabolite remdesivir), the active site cleft of Nsp12 (RdRp) binds to the first turn of gRNA template, while Nsp8 is involved in the formation of sliding poles regulating the processivity of the RdRp (16, 17). The Nsp12 needs to associate with Nsp8 and Nsp7 to activate its capability to replicate long RNA. The Nsp13 helicase is also present in the SARS-CoV-2 replication complex and facilitates the RdRp function (172). Recently, the antiviral activity of CsA was evaluated *in vitro* on Vero E6 cells infected by SARS-CoV-2 and treated 1 h postinfection with serial drug dilutions, and it was reported an anti-SARS-CoV-2 at 50% effective concentration (EC_50_) of 3.5 μM to be compared with 1.5 μM for chloroquine and 5.2 μM for lopinavir (21). Interestingly, the non-immunosuppressive CsA derivative alisporivir (Debio-025), previously reported to inhibit the *in vitro* replication of the human coronavirus HCoV-NL63 (173), was assayed for SARS-CoV-2 inhibition on Vero E6 cells infected for 3 h at a MOI of 0.05 and was found to reduce SARS-CoV-2 production in a dose-dependent manner, with an EC_50_ of 0.46 μM (22). These results suggest that CsA inhibits the viral replicative machinery likely through interaction with a member of the CyP family. Although CyPA depletion so far failed to trigger the reduction of SARS-CoV-1 replication (see above), a function for CyPA in SARS-CoV-2 replication cannot be excluded. It was also previously reported that the transmembrane glycoprotein CD147 (also known as extracellular matrix metalloproteinase inducer EMMPRIN) is facilitating viral replication by interacting with the N protein of SARS-CoV-1 through CyPA (146). CD147 was also reported to bind extracellular CyPB and to stimulate T lymphocytes (174). In COVID-19 patients, the anti-CD147 antibody meplazumab was claimed to improve the recovery of patients, suggesting a role for the CyPA/CD147 complex in SARS-CoV-2 replication similar to that previously described for SARS-CoV-1 (175). Finally, in their very elegant work, Gordon and colleagues set up a SARS-CoV-2 protein interactome map which identified 332 high-confidence protein interactions between SARS-CoV-2 proteins and human cellular compounds. This study revealed that the Nsp2 protein of SARS-CoV-2 interacts with FKBP15 and that the ORF8 of SARS-CoV-2 interacts with FKBP7 and FKBP10 (176). Altogether, these results suggest that CsA acts at different levels in infected cells to prevent the SARS-CoV-2 replication cycle (Figure 6).

## CsA and Cyclophilin in the Renin–Angiotensin System Pathway: Implication for COVID-19

More than two decade ago, it was shown that the formation of abdominal aortic aneurysm in the rat model of elastase infusion was attenuated by CsA treatment (177). CyPA is known to promote atherosclerosis through stimulation of low-density lipoprotein uptake, decrease of endothelial nitric oxide synthase (eNOS) expression, increase of vascular cell adhesion molecule 1 (VCAM-1), and induction of tumor necrosis factor alpha (TNFα) (178). It was reported that deletion of CyPA in mice prevents the formation of abdominal aortic aneurysm in response to infusion of angiotensin II (Ang II) (179).

Although CyPA is an intracellular molecule, it can be secreted from macrophages in response to inflammatory stimuli acting as a chemoattractant of monocytes (63), and it is also secreted by endothelial cells and vascular smooth muscle (VSM) cells and stimulates proinflammatory signals thereby contributing to cardiovascular diseases (180, 181). Extracellular CyPA triggers IκBα phosphorylation that activates the nuclear translocation of NF-κB into the cell nucleus stimulating the transcription of VCAM-1 and E-selectin (66). Indeed, CypA secretion is regulated by Rho-kinase and behaves as a secreted oxidative stress molecule contributing to the pathogenesis of arteriosclerosis, hypertension, and heart failure, and inhibition of Rho-kinase by fasudil reduces the Ang II-induced aortic aneurysm formation (182, 183). Reactive oxygen species (ROS) were found to contribute to the pathogenesis of arteriosclerosis through induction of extracellular signal-regulated kinases ERK1/2 and p38 MAP kinase signaling which stimulated VSM cell growth (184–186). ROS-induced VSM cell growth and proinflammatory signal have been implicated in the revascularization of obstructive coronary artery disease and the pathogenesis of neointima following vascular injury (187). Serum levels of CyPA were found elevated in coronary artery disease (188–190). CypA secreted from blood vessels and heart cells regulates signal pathways and causes a decline of diastolic and systolic function leading to proliferation of cardiac fibroblasts, the occurrence of cardiac hypertrophy, and remodeling (191).

Taniyama and colleagues reported that Ang II activates p38 MAPK inducing an Akt signaling pathway that results in VSM cell activation and suggested that the ROS-sensitive 3-phosphoinositide-dependent protein kinase 1 (PDK1) phosphorylates Akt and that a parallel pathway that requires NADPH oxidase (NOX)-dependent production of ROS (including superoxide anions ${\text{O}}_{2}^{-}$, hydrogen peroxide H_2_O_2_, and hydroxyl radical OH) triggers p38 MAPK activation that in turn activates Akt (186). CyPA was also found to be involved in the translocation of NOX enzymes and the two molecules synergize to increase ROS production (192). Finally, it was also reported that Ang II triggers the release of CyPA and the activation of metalloproteinase 2 (MMP-2) in VSM cells derived from human abdominal aortic aneurysm (62). Ang II type 1 receptor (AT1R) blockers have been shown to prevent cardiovascular diseases (193). During treatment with simvastatin (a member of the statin family which inhibits the hydroxymethylglutaryl CoA reductase), patients with abdominal aortic aneurysm were found to have reduced CypA mRNA expression as well as reduced CyPA intracellular protein levels (194). Interestingly, in a mice model, deletion of the CyPA gene prevented the formation of abdominal aortic aneurysm usually observed in response to infusion of Ang II (179).

**Figure 7:**
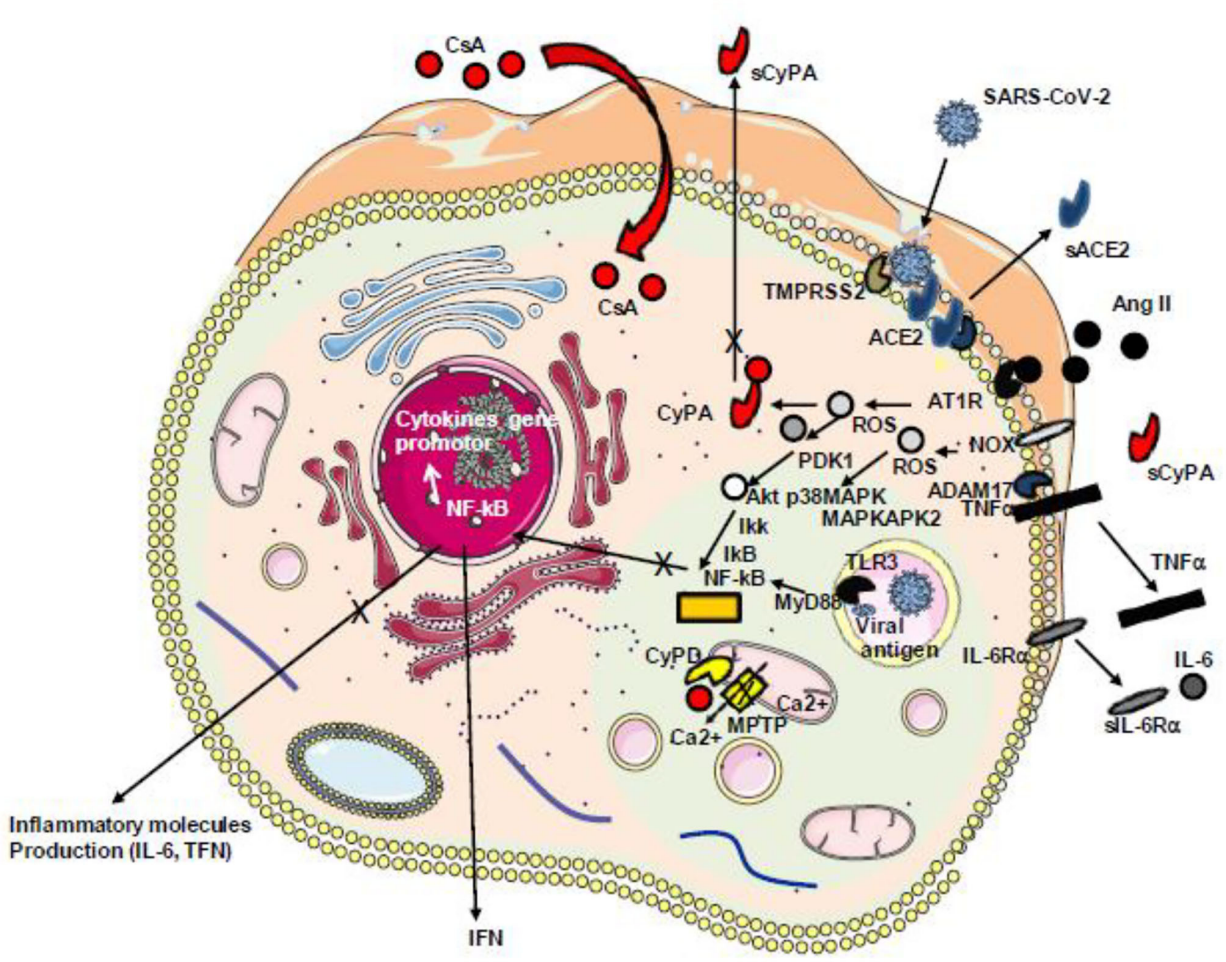
Schematic representation of Ang II/AT1R-induced inflammatory pathway with cytokine release. During infection with SARS-CoV-2, the virus binds ACE2 reducing the ACE2 transcription and inhibiting the capacity of ACE2 to mediate the cleavage of angiotensin II (Ang II) into angiotensin 1–7. The accumulation of Ang II triggers signals through its receptor AT1R inducing ROS production. ROS triggers the secretion of CyPA that acts as a stress factor activating the ERK1/2 kinase and overproduction of ROS through a positive feedback loop. ROS-sensitive 3-phosphoinositide-dependent protein kinase (PDK1) activation that contributes to phosphorylation and activation of Akt. A parallel pathway involves the NOX-dependent generation of ROS that activates the p38 MAP kinase (p38MAPK) which recruits MAPKAPK2 leading to AkT phosphorylation on a second amino acid position leading to full activation of the p38 MAPK–Akt complex, the activation of IKKαβ inducing the release of IkB from the IκB–NF-κB complexes, nuclear translocation of NF-κB, and the production of cytokines including TNF-α and soluble IL-6 receptor (sIL-6R) *via* disintegrin and metalloprotease 17 (ADAM17) followed by the activation of the IL-6 amplifier (IL-6 AMP) which, by feedback regulation, activates both the NF-κB and STAT3 transcription factors and the production of IL-6. SARS-CoV-2 itself activates NF-κB *via* the TLR3 receptor. Ang II, angiotensin II; AT1R, angiotensin II type 1 receptor; ROS, reactive oxygen species; NOX, NADPH oxidase; IKK, IkB kinase; CyPA, cyclophilin A; TLR3, Toll-like receptor 3; NF-κB, nuclear factor κB.

In SARS-CoV-2-infected individuals, the host angiotensin-converting enzyme A (ACE2) monocarboxypeptidase serves as a cell-surface receptor for the virus which interacts with ACE2 by the receptor-binding domain present in its spike (S) protein [reviewed in (195)]. We have recently found evidence that SARS-CoV-2-infected cells have a downregulation of ACE2 mRNA expression and a reduced cell surface expression of ACE2 and that COVID-19 patients have decreased soluble ACE2 and increased levels of Ang II in their plasma (196). Besides the vasoconstrictor and thrombotic effects of Ang II, the dysregulation of the renin–angiotensin pathway with the massive Ang II accumulation is likely to promote the production of proinflammatory cytokine *via* AT1R interaction, by activating metalloprotease 17 (ADAM17) which can process the membrane-anchored TNFα to a soluble TNFα which acts as an activator of NF-KB and IL-6Rα to a soluble form (sIL-6Rα) which can form a complex with IL-6 and activates a STAT3 signaling pathway (197, 198). Since Ang II triggers the release of extracellular CyPA through regulation of Rho-kinase and that extracellular CyPA behaves as a secreted oxidative stress molecule triggering the activation of the NF-κB that stimulates the transcription of VCAM-1 and E-selectin and the overexpression of TNFα the inhibition of CyPA with CsA in COVID-19 patients could reduce atherosclerosis, hypertension, and heart failure. Interestingly, treatment of COVID-19 patients with a recombinant soluble human ACE2 (hrsACE2 from Apeiron Biologics, Vienna, Austria) which can interfere with virus binding but also with Ang II reduced SARS-CoV-2 load and induced a massive decrease of Ang II levels, IL-6, and TNF in patients and showed a strong benefit for the outcome of the patients (199) (Figure 7).

## Conclusion

The emergence of the COVID-19 pandemic about 1 year ago has stressed healthcare systems worldwide, and besides improving the care of patients as knowledge of the disease improves, there was a global race to identify as fast as possible effective drugs to treat SARS-CoV-2-infected patients while waiting to be able to protect individuals with an effective vaccine (200). Since no antiviral was specifically developed against this new coronavirus, the number of clinical trials of molecules expected to interfere with the viral replication cycle or to modulate the immune response has been greater than ever. In this emergency context, the fastest strategy that has been followed by the majority of healthcare teams has been the repositioning of molecules already approved by the US Food and Drugs Administration. Among other molecules, there is ample evidence that CsA may represent a molecule to be tested further in its repurposing therapeutic strategy to treat patients with severe forms of COVID-19. This molecule is widely available, FDA-approved, and affordable. It prevents proinflammatory processes, blocks SARS-CoV-2 replication, and interferes with angiotensin II harmful effects. Recently, Guisado-Vasco et al. (201) reported on the clinical characteristics and outcomes of 607 patients with severe forms of COVID-19 receiving antiviral, antimalarials, glucocorticoids, or immunomodulation with tocilizumab or CsA. From this retrospective observational study (COQUIMA cohort), the authors conclude that among the prescribed therapies, only CsA was associated with a significant (four-fold) decrease in mortality. Moreover, this study adds clear information on the dosing (cumulative dose at least 300 mg) and duration (max 3 weeks) of CsA repurposing in COVID-19.

Therapeutic doses of CsA are usually in the range of 10–20 mg/kg daily when given orally. A wide variability in CsA pharmacokinetics has been observed after the oral or intravenous administration of this drug to patients and varies with respect to the organ grafted, age of the patient, and patient health status. CsA is absorbed in the gastrointestinal tract and almost completely metabolized in both the liver and small intestine by cytochrome P450 family 3 (CYP3A). CsA is also given as intravenous infusion using 2.5–5 mg/kg daily. CsA bioavailability in patients range from 5 to 90%. CsA has the advantage of the intravenous application route which may be crucial for the treatment of critically ill patients with severe forms of COVID-19. However, it is important to emphasize that the serum levels of CsA in conventional treatment fold above the *in vitro* drug concentration required for the inhibition of SARS-CoV-2 replication. The CsA concentration required to inhibit virus replication exceeds the serum concentration of the drug that is usually well below 200 ng/ml (202). A major challenge is to obtain appropriate concentrations of CsA in infected tissues, which will likely require three- to six-fold higher doses than those usually given to patients, which will strongly increase the risks for toxic effects (100). Under these conditions, it is not possible to conclude that the lower COVID-19 mortality reported under CsA treatment is due to an antiviral effect; it could as well result from an anti-inflammatory effect and/or prevention of the deleterious action of Ang II.

Given the variety of side effects of CsA, a careful evaluation of cost/benefit should be done before considering this molecule as a first-line therapy in COVID-19. Nephrotoxicity is the most common adverse effect of CsA treatment and is frequently associated with arterial hypertension (203–205). CsA nephrotoxic effect is dose and duration dependent (206). Vascular effects in the kidney lead to reduced glomerular filtration and impaired sodium excretion. Changes in blood pressure can develop within a few weeks of treatment and sometimes are severe and associated with intracranial hemorrhage, left ventricular hypertrophy, microangiopathic hemolysis, and organ damage (207, 208). This could be a problem as many patients with mild or severe forms of COVID-19 have high blood pressure. In addition, several animal studies have highlighted a vasoconstrictor effect of CsA (209–211). Hypertension and nephrotoxicity must be monitored carefully in patients under CsA therapy. Yet, CsA was reported to protect against Ang II-induced organ damage in transgenic rats harboring human renin and angiotensinogen genes by inhibiting perivascular monocyte/macrophage infiltration and IL-6 and iNOS expression (212). Moreover, many drugs including amphotericin B, aminoglycoside antibiotics, and co-trimoxazole are at risk to potentiate the nephrotoxicity of CsA (202). Indeed, there is a long list of drugs that were proven or suspected to clinically interact with CsA (213) such as anticonvulsants (carbamazepine, phenobarbital, phenytoin, primidone) that reduce CsA blood concentration, antidepressants (fluvoxamine, nefazodone), antimicrobial and antifungal drugs (ketoconazole, fluconazole, itraconazole, metronidazole, fluoroquinolones, macrolides, clarithromycin, erythromycin), antiviral drugs (ritonavir, saquinavir), cardiovascular drugs (amiodarone, calcium channel blockers, amlodipine, nicardipine, verapamil, carvedilol), and hypoglycemic drugs (glibenclamide, glipizide) among others. This list also includes chloroquine and glucocorticoids, which are sometimes used in COVID-19 therapy. The adverse effects of CsA treatment include nephrotoxicity (risk increased by ACE inhibitors among many other drugs), hypertension, hyperkalemia (risk increased by potassium salts), hyperlipidemia, hypomagnesemia, neurotoxicity (risk increased by imipenem), hepatotoxicity (risk increased by androgens), posttransplant diabetes, gingival hyperplasia (risk increased by nifedipine), and hirsutism. Moreover, CsA was reported as able to augment Ang II-stimulated rise in intracellular free calcium in vascular smooth muscle cells (214) and to increase ADAM17 activity up to three-fold, likely leading to an ACE2 shedding increase detrimental to COVID-19 patients (215, 216).

The data in the literature are clear regarding the effects of CsA on *in vitro* SARS-CoV-2 replication, but these are not the only possible beneficial effects one would expect from CsA experimental use in the treatment of COVID-19 since it can modulate both proinflammatory responses and the RAS pathway. Moreover, as summarized in Table 3, several preliminary CsA clinical trials performed on COVID-19 patients are encouraging and suggest that this strategy should be pursued further. In this review, we describe at least three possible mechanisms for which it can be postulated that they are likely to produce a favorable effect on the outcome of COVID-19 patients: (i) an anti-inflammatory effect reducing the production of proinflammatory cytokines, (ii) an antiviral effect preventing the formation of the viral RNA synthesis complex, and (iii) an effect on tissue damage and thrombosis by acting against the deleterious action of angiotensin II. It is also possible that CsA contributes to decrease the lactate/pyruvate ratio in cells by activating the NHE-3 Na^+^/H^+^ exchanger, thereby counteracting the hypoxic damage induced by SARS-CoV-2 infection (215, 217). Even if CsA has many effects that are likely to improve the outcome of patients infected with SARS-CoV-2, one can of course wonder about the consequence of using a therapeutic drug that exhibits immunosuppressive effects in severe forms of COVID-19 because this could reduce the innate and adaptive immune responses of the patients against the virus (146, 218–220). However, there is an increasing panel of available cyclophilin inhibitors such as alisporivir/Debio-025 (Novartis), Debio-064 (Novartis), SDZ NIM811 (Sandoz, Novartis), SCY-635 (Scynexis Inc., Jersey City, NJ, USA), STG-175 (S&T Global, Woburn, MA, USA), CRV431 (Hepion Pharmaceuticals, Edison, NJ, USA) or CPI-431-32 (Ciclofilin Pharmaceuticals Inc., San Diego, CA, USA), and it is still possible to replace CsA by one of these compounds or compare these molecules in clinical trials. Finally, as recently highlighted by Schuurmans and Hage (221), it will be very important to decide when CsA should be administered to SARS-CoV-2-infected patients and what should be the effective cumulative dose based on oral or intravenous CsA administration, to obtain the most beneficial effects. Originally used as salvage therapy in refractory COVID-19 cases, CsA could soon be seen as a first-line therapy in COVID-19.

## Author Contributions

CAD, CM, M-DP-M, and DR contributed to the concept of the study. CM designed the tables. CAD designed the figures and wrote the paper. CD provided the histological data. DR obtained the funding for this study. All authors reviewed and approved the final version of the manuscript.

## Conflict of Interest

CAD declares owning Sanofi and Merck shares. The remaining authors declare that the research was conducted in the absence of any commercial or financial relationships that could be construed as a potential conflict of interest.

## Publisher's Note

All claims expressed in this article are solely those of the authors and do not necessarily represent those of their affiliated organizations, or those of the publisher, the editors and the reviewers. Any product that may be evaluated in this article, or claim that may be made by its manufacturer, is not guaranteed or endorsed by the publisher.
